# Low-FODMAP Diet for Gastrointestinal Symptoms in Endometriosis: A Systematic Review

**DOI:** 10.3390/nu18132164

**Published:** 2026-07-03

**Authors:** Rafał Watrowski, Stoyan Kostov, Sebastian D. Schäfer, Ingolf Juhasz-Böss, Mario Palumbo, Radmila Sparić, Liliana Mereu, Salvatore Giovanni Vitale, Ibrahim Alkatout

**Affiliations:** 1 Department of Gynecology, Helios Hospital Müllheim, Heliosweg 1, 79379 Müllheim, Germany; 2 Faculty of Medicine, University of Freiburg, 79106 Freiburg, Germany; 3 Department of Gynecology, Hospital “Saint Anna”, 9002 Varna, Bulgaria; drstoqn.kostov@gmail.com; 4 Research Institute, Medical University Pleven, 5800 Pleven, Bulgaria; 5 Department of Gynecology and Obstetrics, Clemenshospital Münster, 48153 Münster, Germany; seb.schaefer@alexianer.de; 6 Department of Obstetrics and Gynecology, Medical Center—University Hospital Freiburg, 79106 Freiburg, Germany; ingolf.juhasz-boess@uniklinik-freiburg.de; 7 Department of Public Health, School of Medicine, University of Naples Federico II, 80138 Naples, Italy; mario.palumbo@unina.it; 8 Faculty of Medicine, University of Belgrade, Dr Subotića 8, 11000 Belgrade, Serbia; radmila@rcub.bg.ac.rs; 9 Clinic for Gynecology and Obstetrics, University Clinical Centre of Serbia, Dr Koste Todorovića 26, 11000 Belgrade, Serbia; 10 Obstetrics and Gynecology Unit, “Gaspare Rodolico—San Marco” University Hospital, Department of General Surgery and Medical Surgical Specialties, University of Catania, 95124 Catania, Italy; liliana.mereu@unict.it (L.M.); salvatoreg.vitale@unict.it (S.G.V.); 11 Department of Obstetrics and Gynecology, Medical University of Innsbruck, 6020 Innsbruck, Austria; ibrahim.alkatout@i-med.ac.at

**Keywords:** endometriosis, low-FODMAP diet, FODMAP restriction, gastrointestinal symptoms, endo belly, bloating, irritable bowel syndrome, visceral hypersensitivity, dietary intervention, quality of life

## Abstract

**Background/Objectives:** Gastrointestinal (GI) symptoms, including abdominal pain, bloating, altered stool pattern, dyschezia, and nausea, are frequent in women with endometriosis and may persist despite conventional gynecological treatment. The low fermentable oligosaccharides, disaccharides, monosaccharides, and polyols (low-FODMAP) diet is an established dietary intervention for irritable bowel syndrome. Its endometriosis-specific evidence base remains limited. This systematic review evaluated clinical evidence on the low-FODMAP diet or structured FODMAP restriction for GI symptoms in women with endometriosis. **Methods:** This systematic review was prospectively registered in PROSPERO (CRD420261388786) and conducted according to the Preferred Reporting Items for Systematic Reviews and Meta-Analyses (PRISMA) 2020 statement. PubMed/MEDLINE, EBSCOhost, and BASE were searched from inception to 30 April 2026. Eligible reports were clinical studies investigating low-FODMAP diet or structured FODMAP restriction in women with confirmed, clinically diagnosed, imaging-based, or medically reported endometriosis and extractable GI or related clinical outcomes. Risk of bias was assessed with design-specific tools. Due to substantial heterogeneity across studies in design, comparators, and outcome measures, a narrative synthesis was performed. **Results:** Five clinical reports met the inclusion criteria: one randomized controlled crossover feeding trial, two prospective non-randomized studies, one retrospective audit of prospectively collected clinic data, and one case report. The randomized trial showed greater GI response during a 28-day low-FODMAP feeding period than during a nutritionally matched control diet. Prospective studies reported improvements in selected GI symptoms, constipation, pain, and quality-of-life domains, but interpretation was limited by non-randomized allocation, attrition, and mixed or pooled diet comparisons. The retrospective audit and case report supported clinical plausibility but were hypothesis-generating. **Conclusions:** The five available studies, though limited in number and design, indicate that a low-FODMAP diet can reduce GI symptoms in women with endometriosis, particularly those with abdominal pain, bloating, constipation, or IBS-like symptoms. Currently, the low-FODMAP diet should be viewed as a potentially useful, dietitian-guided GI symptom intervention for selected patients. Future trials should define responder profiles, assess long-term tolerability and nutritional safety, and determine the added value of reintroduction and personalization beyond short-term restriction.

## 1. Introduction

Endometriosis is a chronic estrogen-dependent disease of gynecologic origin with systemic consequences [1,2]. The disease affects approximately one in ten women of reproductive age, often begins in adolescence, and produces a cumulative burden of pain, subfertility, fatigue, reduced work capacity, psychosocial distress, and prolonged healthcare contact [1,2,3].

Pathogenetically, endometriosis is multifactorial and can be described with the “seed-and-soil” metaphor [4]. The retrograde menstruation hypothesis proposed by Sampson approximately 100 years ago describes the route of dissemination for the “supercharged” altered eutopic endometrium, but the complexity of endometriosis pathogenesis includes hormonal and immune dysregulation, peritoneal inflammation, neurogenic inflammation, and peripheral and central sensitization that contribute to chronic pelvic and visceral pain [5,6,7]. The pain phenotype frequently extends beyond cyclic dysmenorrhea to non-cyclic pelvic pain, dyspareunia, and bowel- and bladder-related symptoms, in patterns that map only weakly onto lesion location and stage [6,7,8].

Diagnostic delay of several years to more than a decade is common and contributes to disease progression and accumulating disability [8,9]. Current guidelines have shifted from mandatory histological confirmation toward symptom- and imaging-based diagnosis [10,11,12]. The research on non-invasive biomarkers from saliva [13], serum [14], urine [15], or menstrual effluent [4] is expanding, but still far from clinical application. Standard therapy combines analgesia, hormonal suppression, and surgery [8,10]. Each modality has limitations: surgery is invasive, operator-dependent, and unable to address all components of disease-related morbidity; hormonal suppression controls endometrium-driven pain components but is contraceptive, carries cardiovascular considerations, and does not relieve all symptom domains [7,8,16]. Many patients continue to report bothersome symptoms despite optimized standard care, driving interest in adjunctive non-hormonal strategies for persistent symptoms [7,17,18].

GI symptoms are a frequent component of endometriosis, including abdominal pain, bloating, flatulence, constipation, diarrhea, nausea, painful or urgent defecation, incomplete evacuation, and cyclic fluctuation [19,20]. In a case–cohort study of 109 patients and 65 controls, women with endometriosis had higher severity of abdominal pain, constipation, bloating, flatulence, defecation urgency, incomplete evacuation, impaired psychological well-being, and daily life interference, with only a weak association to lesion localization apart from nausea and vomiting in those with bowel-associated lesions. Combined oral contraceptives or progestins did not reduce intestinal symptoms over follow-up [19]. The symptom burden involves mechanisms beyond mechanical bowel infiltration, including pelvic inflammation, altered bowel function, medication effects, visceral hypersensitivity, and comorbid disorders of gut–brain interaction [19,20,21].

Hansen et al. described a “visceral syndrome” cluster in women with endometriosis comprising non-menstrual abdominal pain, dysuria, dyschezia, altered bowel habit, irregular bleeding, nausea or vomiting, and fatigue [22]. As a result, the same patient often consults multiple specialists—gynecologists, gastroenterologists, urologists, and pain clinicians—although all the symptoms are caused by the same underlying condition [22,23].

Bloating and abdominal distension, often intensifying in the luteal phase and during menstruation and associated with increased intestinal wall sensitivity, have been referred to as “endo belly” [20]. Bloating, weight fluctuation, surgical scars, pain, and impaired body function contribute to body-image distress, food-related anxiety, and disordered-eating concerns in a subset of patients [24].

Endometriosis and irritable bowel syndrome (IBS) share abdominal pain, bloating, altered bowel habit, visceral hypersensitivity, symptom fluctuation, and quality-of-life (QoL) impairment [20,21,25]. Reduced intestinal stretch pain thresholds comparable to those reported in IBS have been documented in endometriosis [20,21]. Women with endometriosis have higher odds of IBS than controls (OR 2.97, 95% CI 2.17–4.06), with a pooled IBS prevalence of 23.4% and individual estimates ranging from 10.6% to 52% [25]. The overlap may reflect true comorbidity, diagnostic misclassification, shared inflammatory and neural pain pathways, or common mechanisms of visceral hypersensitivity [21,25,26], and raises the question whether interventions developed for IBS are relevant to selected patients with endometriosis-associated GI symptoms, a question that requires disease-specific evidence.

The gut microbiome links endometriosis, IBS-like symptoms, and dietary responsiveness. Dysbiosis has been described in both conditions, with proposed effects on intestinal permeability, immune activation, inflammatory signalling, microbial metabolites, estrobolome-mediated estrogen metabolism, and gut–brain axis regulation [20,26]. Although the endometriosis-specific data are still largely associative, they provide a biological rationale for studying interventions that modify luminal substrates and fermentation, reduce osmotic load, and alter microbial products and food-related symptoms [26,27].

The low fermentable oligosaccharides, disaccharides, monosaccharides, and polyols (low-FODMAP) diet restricts short-chain carbohydrates that are poorly absorbed in the small intestine and rapidly fermented by colonic microbiota [28,29,30]. In IBS, randomized trials and network meta-analytic synthesis establish the low-FODMAP diet as an effective dietary intervention for global symptoms and abdominal pain [27,29,31,32]. Implementation follows three phases—restriction, reintroduction, and personalization—with dietitian guidance to preserve nutritional adequacy and avoid prolonged restriction [30,33]. The reintroduction phase remains heterogeneous in real-world practice, with variability in challenge foods, dose escalation, timing, sequencing, and follow-up [33]. Examples of higher-FODMAP foods and common lower-FODMAP alternatives are provided in Table A1; individual tolerance depends on portion size, preparation, baseline diet, and structured reintroduction.

Evidence from IBS provides a specific rationale for studying FODMAP restriction in endometriosis-associated GI symptoms. In diarrhea-predominant IBS, low-FODMAP feeding has been associated with improved colonic barrier structure and function, reduced mast-cell recruitment and activation, lower mast-cell mediator levels, and TLR4-dependent effects linked to fecal lipopolysaccharide [27]. Pre-intervention fecal supernatants induced barrier dysfunction in experimental models, whereas post-intervention samples did not, and lipopolysaccharide removal, TLR4 antagonism, mast-cell stabilization, or mast-cell deficiency each prevented barrier dysfunction [27]. Although direct evidence in endometriosis populations is currently lacking, these mechanisms may explain the symptom overlap in women whose clinical phenotype features bloating, abdominal pain, altered bowel habits, and visceral hypersensitivity.

The nutrition literature on endometriosis remains heterogeneous, with low-quality evidence, variable exposures and outcomes, and limited randomized data. Diet may influence inflammation, oxidative stress, hormone metabolism, symptoms, and quality of life (QoL), while low-FODMAP treatment appears plausible mainly for patients with coexisting endometriosis and IBS-compatible symptoms [34,35,36]. In this population, restrictive interventions require evaluation against feasibility, adherence, nutritional adequacy, psychological safety, and long-term personalization [24,33,34].

This systematic review summarizes the evidence on whether a low-FODMAP diet or FODMAP restriction improves GI symptoms, bloating or abdominal distension, abdominal or bowel-related pain, IBS-like symptom burden, pelvic pain, dysmenorrhea, QoL, and patient-reported symptom burden in women with endometriosis. Secondary outcomes include stool pattern, constipation, diarrhea, dyspareunia, adherence, adverse effects, acceptability, and patient satisfaction. GI outcomes are separated from gynecologic pain outcomes where the primary studies permit, and controlled, uncontrolled, retrospective, and single-case evidence are interpreted according to their methodological strength.

## 2. Materials and Methods

### 2.1. Protocol Registration and Reporting Standard

This systematic review was prospectively registered with the International Prospective Register of Systematic Reviews (PROSPERO; CRD420261388786). The review was designed and reported in accordance with the Preferred Reporting Items for Systematic Reviews and Meta-Analyses (PRISMA) 2020 statement [37]. The PRISMA 2020 checklist is provided in Supplementary Material.

### 2.2. Eligibility Criteria

Clinical studies were eligible if they investigated a low-FODMAP diet or structured FODMAP restriction, either as a primary intervention or as part of a stepwise dietetic pathway, in adolescent or adult women with surgically confirmed, imaging-based, clinically diagnosed, or participant-reported prior endometriosis diagnosis accepted by the source study protocol, and reported at least one outcome relevant to GI symptoms, pain, QoL, adherence, nutritional adequacy, acceptability, or adverse effects. Endometriosis ascertainment included laparoscopic confirmation, transvaginal sonographic or magnetic resonance imaging (MRI)-based diagnosis, physical examination or clinical diagnosis by a treating physician, or participant-reported prior diagnosis when this was accepted by the original study protocol, in line with diagnostic pathways recognized in current guidelines [10,11,12]. Eligible designs included randomized and non-randomized controlled trials, prospective and retrospective cohort studies, and individual case reports with structured intervention detail. Survey-based dietary-practice studies that did not isolate a structured low-FODMAP intervention with extractable endometriosis-specific outcome data were excluded from the evidence tables. Editorials, narrative reviews, letters without primary data, guidelines, protocols, conference abstracts without sufficient methodological detail, and animal or in vitro reports were excluded. No language restrictions were applied.

### 2.3. Information Sources and Search Strategy

A systematic literature search was conducted in PubMed/MEDLINE, the EBSCOhost platform (including Academic Search Premier, APA PsycArticles, APA PsycInfo, CINAHL, and MEDLINE), and the Bielefeld Academic Search Engine (BASE), from database inception to 30 April 2026. The PubMed search combined MeSH and free-text terms for endometriosis with free-text terms for FODMAPs and related fermentable-carbohydrate concepts:

(“Endometriosis”[Mesh] OR endometriosis[tiab] OR endometrioma*[tiab]) AND (FODMAP*[tiab] OR “low FODMAP”[tiab] OR “low-FODMAP”[tiab] OR “fermentable oligosaccharides”[tiab] OR “fermentable oligo-saccharides”[tiab] OR “fermentable carbohydrate”[tiab] OR “fermentable carbohydrates”[tiab] OR “short-chain carbohydrate”[tiab] OR “short-chain carbohydrates”[tiab] OR “short chain carbohydrate”[tiab] OR “short chain carbohydrates”[tiab] OR (oligosaccharides[tiab] AND disaccharides[tiab] AND monosaccharides[tiab] AND polyols[tiab])).

The EBSCOhost search applied a parallel construction across MH (subject heading), TI (title), AB (abstract), and TX (all text) fields:

(MH “Endometriosis+” OR TI (endometriosis OR endometrioma*) OR AB (endometriosis OR endometrioma*)) AND (TI (FODMAP* OR “low FODMAP” OR “low-FODMAP”) OR AB (FODMAP* OR “low FODMAP” OR “low-FODMAP”) OR TX (“fermentable oligosaccharides” OR “fermentable oligo-saccharides” OR “fermentable carbohydrate” OR “fermentable carbohydrates” OR “short-chain carbohydrate” OR “short-chain carbohydrates” OR “short chain carbohydrate” OR “short chain carbohydrates”) OR (TX oligosaccharides AND TX disaccharides AND TX monosaccharides AND TX polyols)).

The BASE search used the all-fields query *“Low-FODMAP” AND “Endometriosis”*. Reference lists of all included reports and relevant systematic reviews were screened for additional records.

**Figure 1:**
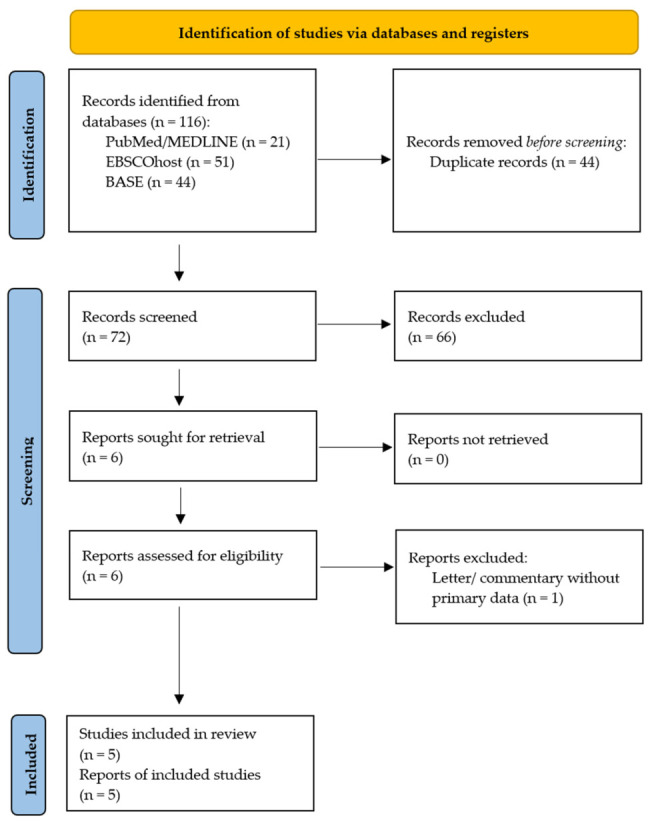
PRISMA 2020 flowchart of study identification and selection.

The PubMed/MEDLINE search retrieved 21 records. The EBSCOhost search retrieved 51 records, of which 26 were duplicates of PubMed/MEDLINE records, and none represented an additional eligible study. The BASE search retrieved 44 records; after consolidation of 18 repeated BASE entries referring to the same reports and subsequent eligibility screening, BASE contributed one additional included report [38]. The selection process is summarized in the PRISMA 2020 flow diagram (Figure 1).

### 2.4. Study Selection

Two reviewers (R.W., S.K.) independently screened titles and abstracts and subsequently assessed full-text reports against the eligibility criteria. There were no disagreements. No automation tools were used. Five primary clinical reports met the inclusion criteria and were retained for descriptive synthesis [38,39,40,41,42].

### 2.5. Data Extraction

Data were extracted directly from the full texts of the included studies into a structured evidence table by one reviewer and verified by a second. Extracted variables were organized into the following categories: (1) study metadata (authors, year, journal, setting, registration); (2) population characteristics (sample sizes at consent, start, and completion; age; menopausal status; diagnostic basis for endometriosis; Rome criteria and IBS comorbidity); (3) intervention and comparator details (follow-up duration; low-FODMAP protocol specifics such as restriction duration, target intake, food provision versus advice, lactose handling, and masking; dietitian involvement; educational materials; reintroduction and personalization protocols); (4) outcomes and instruments (primary and secondary definitions; GI and pain outcomes, including endometriosis-specific tools like EHP-30/23; GI-specific QoL via Gastrointestinal Quality of Life Index (GIQLI); psychological outcomes via DASS-21 and equivalents); and (5) adherence and safety (assessment methods and results, dropout reasons, acceptability, post-study continuation, and adverse events). Where reported, substrate-specific reintroduction tolerance was extracted using the original substrate identifiers and dose categories. Numerical values are reported in their original metric.

### 2.6. Risk-of-Bias Assessment

Risk of bias was assessed using tools matched to the study design. The revised Cochrane risk-of-bias tool for randomized trials (RoB 2) [43], with the additional considerations for crossover trials, was applied to the EndoFOD trial [39]. The Risk Of Bias In Non-randomized Studies of Interventions tool (ROBINS-I) [44] was applied to the prospective cohort study by Keukens et al. [40], the prospective patient-preference pilot study by van Haaps et al. [41], and the retrospective audit by Moore et al. [42]. The Joanna Briggs Institute critical appraisal checklist for case reports [45] was applied to the case report by Jankovich and Watkins [38]. Risk-of-bias judgements were performed independently by two reviewers and reconciled by discussion with a third reviewer. Risk-of-bias-relevant limitations, methodological strengths, and the resulting overall judgement for synthesis are presented in Table A2.

### 2.7. Data Synthesis

A quantitative meta-analysis was not appropriate given substantial heterogeneity in study design, control condition, intervention duration, low-FODMAP protocol implementation, endometriosis ascertainment, IBS comorbidity criteria, follow-up, and outcome instruments. The evidence was synthesized narratively. Findings were organized by populations and clinical contexts, diet implementation and adherence, GI outcomes, including global response, bloating and abdominal distension, and stool pattern, pain and endometriosis-specific QoL outcomes, GI-specific QoL and psychological outcomes, acceptability and safety, and risk-of-bias-relevant limitations. The synthesis distinguishes restriction-phase data from reintroduction- and personalization-phase data. Only the randomized crossover feeding trial isolated the short-term restriction phase under controlled dietary conditions. The non-randomized prospective studies included reintroduction and personalization components but did not allow phase-specific causal attribution. Effect estimates from individual studies are reported in the original metric without recoding or post hoc imputation. Where studies separated low-FODMAP-arm-specific from pooled-diet effects, both estimates are reported, and the limits on causal attribution are stated explicitly.

## 3. Results

Five primary clinical reports met the inclusion criteria and were retained for descriptive synthesis: one randomized controlled crossover feeding trial [39], one prospective single-centre cohort with a structured elimination and reintroduction pathway [40], one prospective patient-preference pilot study with a parallel no-diet control arm [41], one retrospective audit of a private community-based IBS clinic with a laparoscopically defined endometriosis subgroup [42], and one structured single-patient case report from a private dietetic practice [38]. Study designs, eligibility criteria, comparators, and outcome instruments are summarized in Table 1, protocol implementation and adherence in Table 2, GI outcomes in Table 3, pain, QoL, acceptability and safety in Table 4, and risk-of-bias-relevant limitations together with the overall judgement for synthesis in Table A2.

### 3.1. Populations and Settings

The included studies varied substantially in sample size and recruitment context (Table 1). The largest cohort, by Moore et al., retrospectively analyzed 160 women meeting Rome III criteria for IBS at a single private clinic, 59 of whom had concurrent laparoscopically diagnosed endometriosis [42]. At the other end of the spectrum, Jankovich and Watkins provided a detailed single-case report of a 23-year-old woman with laparoscopically treated ovarian endometriosis and IBS [38]. Among the prospective studies, Varney et al. randomized 35 women with a prior diagnosis of endometriosis and a baseline overall GI VAS score above 30 mm; notably, Rome criteria were deliberately not required, and 25 women completed both diet periods [39]. Keukens et al. enrolled 47 premenopausal adults with debilitating bowel symptoms, though this study required a clinical diagnosis of endometriosis (via examination, imaging, or laparoscopy), and 58.3% of completers met Rome III criteria for IBS at baseline [40]. Van Haaps et al. took a different approach, using a patient-preference design with 62 per-protocol participants who self-selected into a low-FODMAP arm (*n* = 22), an endometriosis-targeted diet arm (*n* = 21), or usual care (*n* = 19); eligibility required a pain score of at least 3 out of 10 despite ongoing medical treatment [41].

### 3.2. Diet Implementation and Adherence

Implementation, dietetic input, and reintroduction structure varied widely across studies (Table 2). The EndoFOD trial used a fully supplied feeding design with a low-FODMAP arm below 5 g/day FODMAPs and a control arm of approximately 20 g/day FODMAPs, both modelled on the Australian Dietary Guidelines and matched for energy, macronutrients, fibre, gluten exposure, and low lactose content. Daily adherence was 75% on low-FODMAP days versus 71% on control days, with dropouts of 2 during diet 1, 2 during washout, and 6 during diet 2 [39]. Keukens et al. used a 4-week strict elimination followed by an at least 10-week dietitian-guided reintroduction, with a mean non-adherence of 8% in completers and 50% choosing to continue the diet after study end; the 13 of 47 pre-start withdrawals and the 10 of 34 post-start dropouts together constrain the analysis to motivated completers [40]. Van Haaps et al. delivered three 1 h and three 30 min consultations during the 3-month guided phase, followed by 3 months of independent continuation; 81.8% of low-FODMAP participants and 35 of 43 across both diet arms wished to continue at least partially [41]. Moore et al. taught a single low-FODMAP regimen to all Rome III patients in the clinic, with adherence captured by direct questioning, reaching 55 of 59 (93%) in the endometriosis subgroup and 91 of 101 (90%) in the IBS without known endometriosis subgroup [42]. Jankovich and Watkins applied a stepwise 16-week pathway with two individual and two group meetings, including a 2-week first-line dietary and lifestyle phase, a 6-week strict elimination including lactose, and a structured 10-week reintroduction; the substrate-specific tolerance map at full clinical re-challenge showed full-dose tolerance for lactose, sorbitol and galacto-oligosaccharides, no tolerance for mannitol and garlic fructans, minimal tolerance for fructose and wheat fructans at half of the initial challenge dose, and tolerance of the initial dose for onion fructans [38].

### 3.3. GI Outcomes

GI outcomes generally favoured symptom improvement after low-FODMAP restriction or dietitian-guided dietary intervention, although the strength of evidence differed by design and comparator (Table 3). In the EndoFOD trial, responders were defined by a decrease of more than 20 mm in the 100 mm overall GI visual analogue scale (VAS) from baseline to the end of the dietary intervention and/or an end-of-intervention score below 30 mm. In intention-to-treat analysis, 21 of 35 participants (60%) responded to the low-FODMAP diet versus 9 of 35 (26%) to the nutritionally matched control diet (*p* = 0.008). Per-protocol responders were 18 of 25 (72%) versus 8 of 25 (32%) (*p* = 0.01), and week-4 overall GI symptom scores were 35 mm versus 58 mm (*p* < 0.001). Patient-Reported Outcomes Measurement Information System GI (PROMIS-GI) bloating T-scores improved on the low-FODMAP diet versus baseline and control, normal stool form was recorded on 71% of low-FODMAP days against 43% at baseline and 56% on control, and PROMIS-GI diarrhea improved versus control [39]. Keukens et al. reported a primary outcome of decreased constipation after reintroduction, with the Agachan Constipation Scoring System falling from a median of 7.0 (interquartile range 5) to 5.0 (interquartile range 4), giving a mean difference of 2.1 (95% confidence interval (CI) 0.4 to 3.7, *p* = 0.023). Binary bloating prevalence did not change significantly; the transient decrease after elimination was non-significant (*p* = 0.125), and all respondents with post-reintroduction data again reported bloating. Separately, 84% of completers reported decreased bowel symptoms, and 53% of completers reported less bloating after the diet [40]. In van Haaps et al. [41], the diet groups combined showed less bloating than controls over 6 months (mean difference −0.84, 95% CI −1.68 to −0.004, *p* = 0.049), but the separated low-FODMAP versus control comparison for bloating was not significant (mean difference −0.69, 95% CI −1.66 to 0.27, *p* = 0.159). Within the low-FODMAP arm, bloating improved from baseline (*p* < 0.001), and dysuria improved from baseline (*p* = 0.015). Moore et al. recorded a greater than 50% abdominal symptom reduction in 43 of 59 (72%) women with endometriosis and IBS versus 49 of 101 (49%) patients with IBS without known endometriosis (*p* = 0.001; odds ratio (OR) 3.11, 95% CI 1.5 to 6.2) [42]. In the case study [38], bloating decreased from 8 of 10 to less than or equal to 3 of 10 and abdominal pain from 7 of 10 to less than or equal to 3 of 10 after the 6-week elimination, together with a transition from Bristol type 1 to 2 stools every 2 to 3 days at baseline to type 3 stools every 1 to 2 days at final follow-up.

### 3.4. Pain, QoL, Acceptability and Safety

Endometriosis-specific pain and QoL outcomes were assessed most extensively in the randomized feeding trial [39] and the prospective cohort study [40]. The controlled patient-preference evaluation [41] provided narrower comparative data, whereas the retrospective audit [42] and the case report [38] included limited or no validated assessment of these domains (Table 4).

In the EndoFOD trial, Endometriosis Health Profile-30 (EHP-30) pain improved on low-FODMAP versus control, with a median of 22.7 versus 40.9 (*p* = 0.005). EHP-30 total scores were lower on low-FODMAP than on control, 45.3 versus 47.1 (*p* = 0.028), and the control and powerlessness subdomain improved nominally (*p* = 0.045); GIQLI overall health-related QoL improved on low-FODMAP versus control with a median of 88 versus 81 (*p* = 0.003), with parallel gains in GIQLI symptoms and physical function. According to the Depression Anxiety Stress Scales-21 (DASS-21), depression and anxiety did not differ across diets in the full intention-to-treat analysis. Among participants with abnormal baseline scores, depression decreased on low-FODMAP and anxiety showed a trend; stress decreased versus baseline, but no diet-specific difference was observed in the abnormal-stress subgroup. Pelvic pain, dysmenorrhea and dyspareunia were not assessed as specific symptom endpoints. One serious adverse event of appendicitis occurred during the washout after the control diet and was considered unlikely to be related to the intervention; two participants developed gastroenteritis-like symptoms during the control diet, with one later withdrawing, citing life stress [39]. Keukens et al. reported an EHP-30 pain improvement from 47.8 +/− 20.1 to 29.2 +/− 17.3 (*p* = 0.002), with significant gains across control and powerlessness (69.4 to 36.7, *p* < 0.001), emotional well-being (45.2 to 29.2, *p* = 0.001), social support (46.4 to 31.3, *p* = 0.017), self-image (51.2 to 40.5, *p* = 0.035), work life (35.0 to 21.7, *p* = 0.003), and sexual intercourse (61.6 to 45.7, *p* = 0.023). Of those who started the diet, 63% rated it as easy to maintain. Among completers, 65% reported less pain. Across all participants, 87% described the diet as a good addition to current therapies, and 90% said they would recommend it; no harmful effects were reported [40]. Van Haaps et al. observed within-arm low-FODMAP improvements in dysuria and bloating from baseline and a separated low-FODMAP versus control reduction in deep dyspareunia (mean difference −1.15, 95% confidence interval (CI) −2.2 to −0.10, *p* = 0.032), together with an EHP-30 medical-profession improvement (mean difference −17.14, *p* = 0.018). Dysmenorrhea, chronic pelvic pain, and GIQLI did not differ significantly between the separated low-FODMAP and control arms [41]. Moore et al. [42] did not capture endometriosis-specific QoL or post-diet pain endpoints, and Jankovich and Watkins [38] reported no validated QoL or gynecological pain endpoints, although improved symptom control and trigger identification were documented at one-month follow-up after final consultation.

### 3.5. Risk-of-Bias

The five reports differ substantially in their methodological rigour (Table A2). The EndoFOD trial represents the highest methodological quality, with prospective registration, nutritionally matched supplied diets, menstrual-cycle-controlled timing, validated instruments, and a prespecified responder definition, but it is limited by single blinding without formal assessment of blinding effectiveness, subjective outcomes, a 10 of 35 dropout rate, single-centre Melbourne recruitment via advocacy channels, absence of original diagnostic reports, deliberate non-requirement of Rome criteria, a control FODMAP dose that may exceed habitual intake, no mechanistic biomarkers, and no reintroduction or long-term follow-up [39]. The cohort study [40] is most informative for real-world feasibility, acceptability, and within-subject symptom change among motivated completers, but lacks a control group, randomization, and blinding, with high attrition before and after diet initiation and completer-based efficacy estimation. The controlled pilot study [41] adds a no-diet comparison, but patient-preference allocation introduces selection bias; the diet arms were often pooled, no sample size calculation was performed, no follow-up extends beyond 6 months, and low-FODMAP-specific causal interpretation is limited. The audit study [42] identifies a candidate subgroup in a private clinic, but is limited by restriction to follow-up returners, absence of a placebo or control diet, use of a non-validated 40-item questionnaire, and a comparator group without systematic exclusion of endometriosis by laparoscopy or imaging. The case report [38] illustrates clinical implementation, but has no control, uses a non-validated practice symptom score, and includes concurrent first-line dietary, lifestyle, laxative, probiotic, and stress modifications. Notably, the patient had previously self-reported lactose intolerance after an unstructured home challenge, whereas structured clinical re-challenge later demonstrated full-dose lactose tolerance.

### 3.6. Synthesis-Level Interpretation

Across studies, GI outcomes generally improved after low-FODMAP restriction or dietitian-guided dietary intervention (Table 3). The EndoFOD trial is particularly informative because it compared a supplied low-FODMAP diet directly with a nutritionally matched control diet (ca. 20 g/day FODMAPs), giving the control condition greater dietary specificity than standard dietary advice [39]. Endometriosis-specific QoL and pain outcomes were most extensively assessed in the randomized feeding trial [39] and the prospective cohort study [40]. The controlled patient-preference evaluation [41] added narrower comparative data for deep dyspareunia and the Endometriosis Health Profile-30 (EHP-30) medical-profession domain, while dysmenorrhea, chronic pelvic pain, and GIQLI were not significantly improved in the separated low-FODMAP versus control analysis (Table 4). Evidence on reintroduction and personalization remains sparse and comes from non-randomized or case-report-level evidence. The only randomized controlled trial specifically tested the supplied restriction phase only (Table 2). The EndoFOD trial can therefore be interpreted as the methodologically strongest source for short-term restriction-phase efficacy, Keukens et al. [40] and van Haaps et al. [41] as the principal sources of dietitian-guided real-world feasibility and patient-reported outcomes, Moore et al. [42] as a candidate-subgroup identifier, and the case report [38] as an illustration of structured reintroduction and tolerance testing (Table A2).

## 4. Discussion

Five clinical reports met the inclusion criteria and generally favoured improvement of GI symptoms after low-FODMAP restriction or dietitian-guided FODMAP reduction, with different levels of causal interpretability across designs. The EndoFOD trial provides the most direct low-FODMAP-specific evidence, as its 28-day supplied intervention was tested against a nutritionally matched control diet and showed higher responder rates and lower overall GI symptom scores [39]. The two prospective non-randomized studies extend the evidence toward dietitian-guided practice, acceptability, QoL, and symptom evolution beyond short restriction, although causal attribution is limited by uncontrolled or patient-preference allocation, attrition, and pooled diet-arm analyses in one study [40,41]. The retrospective audit and the case report add clinical plausibility and implementation detail, but their designs cannot separate diet effects from selection, expectation, clinical contact, or co-interventions [38,42].

Women with endometriosis report higher rates of abdominal pain, bloating, constipation, urgency, incomplete evacuation, nausea, fatigue, and impaired daily functioning than controls [19,22]. The term “endo belly” further describes cyclic or peri-menstrual abdominal distension as a clinically relevant symptom, cautioning against reducing all endometriosis-associated bloating to IBS [20]. FODMAP restriction is therefore best understood as a GI symptom-directed intervention in women with endometriosis, not as a treatment for endometriosis itself.

In gastroenterology, the low-FODMAP diet in IBS is supported by a large evidence base. A recent network meta-analysis of dietary interventions in IBS included 28 randomized controlled trials with 2338 patients and confirmed that the low-FODMAP diet was superior to the habitual diet for global IBS symptoms, abdominal pain, and abdominal bloating or distension, although confidence in many network comparisons remained limited [46]. Dietary approaches in this field are not interchangeable as they target different endpoints, including global-symptom burden, abdominal pain, bloating, stool pattern, and inflammatory or quality-of-life measures, and are therefore difficult to compare on any single outcome [46]. In endometriosis specifically, the diet literature remains heterogeneous in exposures and outcomes, and global dietary patterns such as the Mediterranean diet have been proposed as a safer long-term basis, with FODMAP restriction reserved as a symptom-directed option for patients with IBS-like complaints [35,47]. A low-FODMAP diet represents therefore one GI symptom-directed option among several, chosen according to phenotype, feasibility, and nutritional and psychological safety. FODMAPs may increase small-intestinal water content and undergo colonic fermentation, thereby promoting luminal distension, pain, bloating, and stool disturbance in susceptible patients [27,29]. In endometriosis, this rationale is plausible because IBS-like complaints, visceral hypersensitivity, and chronic pelvic pain frequently overlap, but the Rome criteria have not been validated specifically for women with endometriosis [39]. The endometriosis nutrition literature also stresses that restrictive diets may expose patients to nutritional and psychological burden, whereas global dietary patterns may provide a safer long-term basis, and targeted low-FODMAP intervention may be reserved for selected patients with IBS-compatible digestive symptoms [47].

The included studies support a GI symptom-specific interpretation of FODMAP restriction and do not establish a disease-modifying effect in endometriosis. The controlled feeding design in [39] is well suited to assessing short-term fermentable-carbohydrate restriction in women with endometriosis and poorly controlled GI symptoms, but is hardly applicable to patients whose dominant complaints are dysmenorrhea, dyspareunia, infertility, fatigue, or non-GI pain [39]. In the real-world cohort by Keukens et al., improvements in constipation and several EHP-30 domains were observed among completers with debilitating bowel symptoms, although the absence of a control group leaves motivation, regression to the mean, dietitian contact, and symptom fluctuation unresolved [40]. The 6-month controlled pilot supports cautious interpretation because several main analyses pooled low-FODMAP and endometriosis-diet arms. Bloating improved within the low-FODMAP arm, while the separated low-FODMAP-versus-control comparison was not significant [41].

The most plausible clinical candidates are patients with prominent bloating, abdominal pain, altered stool form, constipation, diarrhea, urgency, or IBS-like symptom clusters. The audit by Moore et al. is consistent with this selection principle, as women with Rome III IBS and laparoscopically diagnosed endometriosis had higher bowel-symptom response rates after low-FODMAP instruction than women with IBS without known endometriosis [42]. This comparison remains limited by the lack of systematic screening for endometriosis in the comparator group and by the inclusion of follow-up returners only.

The main biological rationale for low-FODMAP is a reduction in osmotic load, fermentation, gas production, luminal distension, and stimulation of visceral pain pathways. The included endometriosis studies did not measure breath hydrogen or methane, intestinal permeability, inflammatory biomarkers, microbiome composition, or metabolomic change. Among potentially transferable mechanisms are toll-like receptor 4-, lipopolysaccharide-, and mast-cell-dependent epithelial barrier pathways recently characterized in diarrhea-predominant IBS, in which low-FODMAP feeding reduced fecal lipopolysaccharide load and prevented mast-cell-driven barrier dysfunction in experimental models [27]. Zhao et al. therefore correctly noted that mechanistic claims in the EndoFOD context remain inferential without physiological endpoints and that future studies should include objective measures if they aim to link symptom response with fermentation, barrier function, microbiome change, or inflammation [48].

The broader IBS microbiome literature argues against prolonged unguided restriction. Low-FODMAP restriction can improve symptoms but may reduce *Bifidobacterium* and alter microbial composition during the restrictive phase [49]. This concern is relevant to endometriosis because many patients already experiment with gluten-free, dairy-free, “anti-inflammatory”, low-histamine, low-nickel, or other elimination diets, often without dietetic supervision [47]. This mirrors observations in celiac disease, where FODMAP restriction has been proposed to address persistent IBS-like symptoms, while gluten avoidance remains first-line therapy [50]. When implemented, a low-FODMAP diet should be structured, time-limited, professionally supervised, and followed by reintroduction when restriction results in a clinical improvement.

The broader endometriosis-specific diet literature further complicates a blanket application of an IBS-derived low-FODMAP model. Onion and garlic are commonly restricted during the low-FODMAP phase because of their fructan content, but both belong to food groups with experimental or clinical endometriosis-specific data: quercetin inhibited endometriosis-cell proliferation through cyclin-D1- and microRNA-related pathways, high-dose *Allium cepa* reduced proliferative features and Ki67 expression in rat endometriotic implants, and garlic tablets reduced pelvic pain, back pain, dysmenorrhea, and dyspareunia in a randomized placebo-controlled trial [51,52,53]. S-allyl-L-cysteine, a major aged-garlic constituent, also inhibited lesion growth in a mouse model and modulated adhesion-, apoptosis-, and immune-inflammatory pathways, while aged-garlic extract has been proposed as a prophylactic candidate on mechanistic grounds [54,55]. These data argue against presenting low-FODMAP restriction as an intrinsically anti-endometriosis diet. If a low-FODMAP diet is applied, temporary restriction of onion and garlic remains reasonable during the restriction phase to reduce fructan-related symptom provocation. High-FODMAP foods with plausible endometriosis-relevant bioactivity should therefore be treated as context-dependent restriction targets: they may be temporarily restricted during a defined GI symptom trial but should be systematically reintroduced when response permits.

Reintroduction and personalization remain insufficiently studied in the endometriosis-specific context. As the sole randomized trial examined only the 28-day restriction phase, it demonstrates short-term symptom control but does not address long-term dietary self-management [39]. The two prospective non-randomized studies incorporated reintroduction and personalization components, but their designs do not allow phase-specific causal attribution [40,41]. The case report illustrates the clinical role of structured re-challenge, as supervised lactose testing did not confirm prior self-reported dairy intolerance after an unstructured home challenge and helped identify tolerated and poorly tolerated FODMAP subgroups in the individual patient [38].

Safety and acceptability were reassuring but incompletely assessed. No diet-related serious safety concern was reported in the randomized trial, and no side effects were reported in the controlled patient-preference study [39,41]. Feasibility was more complex in the prospective cohort, where 13 of 47 participants withdrew before starting, mostly because of motivation or time constraints, and 10 of 34 discontinued after starting, although completers reported low average non-adherence and high satisfaction [40]. These data suggest good feasibility among motivated completers, with lower certainty for the broader eligible population. Longer follow-up is needed because the included studies do not adequately assess nutritional adequacy, food-related QoL, microbiome effects, or sustainability.

In IBS cohorts, validated screening tools have detected possible eating disorders in approximately one-fifth to one-quarter of patients, with avoidant/restrictive food intake disorder presentations in up to one-third of neurogastroenterology referrals [56,57], and reported prevalence ranging more widely depending on the screening instrument applied [58]. In women with endometriosis, screening studies point to roughly one-third for possible eating disorders and close to half for concurrent IBS-compatible symptoms, alongside a near-threefold genetic liability to eating disorders independent of body mass index, chronic pain, and IBS [59]. Chronic pain with cyclic GI exacerbation, body-image distress related to bloating, weight fluctuation and surgical scars, and food-related anxiety from repeated symptom-trigger learning may further raise the risk of disordered eating in this population [24,56,59]. None of the included studies screened for eating-disorder symptoms before or during intervention or reported food-related QoL as an endpoint. When low-FODMAP intervention is offered, reasonable safeguards include pre-intervention screening with a validated instrument such as Sick, Control, One stone (14 lb/6.35 kg), Fat, Food eating-disorder screening questionnaire (SCOFF) or the nine-item avoidant/restrictive food intake disorder (ARFID) screen, attention to unintended weight loss, and avoidance of unsupervised indefinite restriction [56,57,58,59].

The EndoFOD trial strengthened the evidence base by testing FODMAP restriction under randomized, controlled feeding conditions. Its supplied meals, nutrient matching, menstrual-cycle timing, validated GI and QoL instruments, and prespecified responder definition strengthen internal validity [39]. The limitations discussed by Zhao et al. include small sample size, attrition, Melbourne-only recruitment related to food delivery, recruitment through advocacy networks, limited cultural and dietary diversity, exclusion of women without English proficiency or with irregular cycles, single blinding without formal assessment of blinding success, subjective outcomes, absence of mechanistic biomarkers, and possible carryover effects despite washout [48].

The non-randomized studies provide valuable data on feasibility, selection effects, and clinical applicability. Dietitian-guided pathways appeared acceptable for motivated completers, but attrition before or after diet initiation shows that the intervention can be demanding even when professional support is available [40]. The controlled patient-preference design extends observation to 6 months, although self-selection into the diet arms limits causal attribution because motivation and expectation were probably unevenly distributed between groups [41]. The retrospective IBS-clinic audit contributes a clinically relevant comparison between women with IBS and known endometriosis and women with IBS without known endometriosis, but its comparator cannot be treated as a systematically screened endometriosis-negative group [42].

Future trials should define whether low-FODMAP restriction reduces symptoms, which patients benefit, how long improvement persists, and under which dietary conditions the intervention remains feasible. Parallel-group designs may be preferable when durable dietary and microbial adaptations are expected. Comparators should include active dietary alternatives, such as Mediterranean, anti-inflammatory, gluten-free, or simplified FODMAP-focused approaches, alongside habitual-diet or no-diet controls, since head-to-head IBS evidence shows comparable global-symptom responder rates across some approaches with potentially lower implementation burden [46]. A recent RCT-focused review across GI disorders similarly emphasized heterogeneous protocols, variable outcome measures, the need for dietitian supervision, and caution against treating low-FODMAP restriction as broadly indicated [32]. Baseline phenotyping should separate bowel lesion status, IBS criteria, constipation- versus diarrhea-predominant symptoms, hormonal treatment, opioid use, prior surgery, menstrual suppression, bloating severity, and “endo belly” pattern. Future pooling would be more reliable if trials used a shared minimum outcome set, including a validated global GI response measure, an endometriosis-specific pain assessment such as the EHP-30 pain domain, a structured dyspareunia visual analogue scale, dietary adherence, nutritional adequacy, food-related QoL, adverse effects, and long-term continuation. Cost and access also require assessment, because gluten-free and low-FODMAP-compatible products are often more expensive than standard counterparts, while access to dietitian support varies across health systems [56].

**Figure 2:**
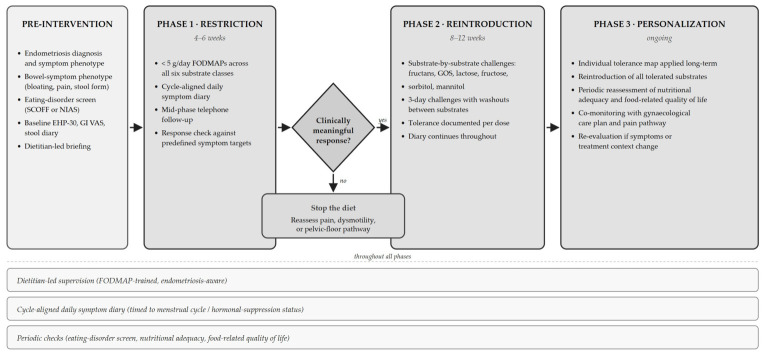
Three-phase low-FODMAP intervention pathway. Phase 1 implements restriction below 5 g/day FODMAPs for 4–6 weeks; on clinically meaningful response, Phase 2 (8–12 weeks of substrate-by-substrate reintroduction) and Phase 3 (long-term tolerance with gynecological co-monitoring) follow; otherwise restriction is stopped and the symptom pathway is reassessed. Pre-intervention assessment, eating-disorder screening (SCOFF or NIAS), cycle-aligned symptom diary, and periodic checks of nutritional adequacy and food-related QoL apply throughout under dietitian-led supervision. Abbreviations: EHP-30, Endometriosis Health Profile-30; FODMAP, fermentable oligosaccharides, disaccharides, monosaccharides, and polyols; GI, gastrointestinal; GOS, galacto-oligosaccharides; NIAS, nine-item avoidant/restrictive food intake disorder screen; SCOFF, Sick, Control, One stone (14 lb/6.35 kg), Fat, Food eating-disorder screening questionnaire; VAS, visual analogue scale.

Breath testing, stool microbiome profiling, fecal metabolomics, inflammatory markers, intestinal permeability measures, and menstrual-cycle-linked symptom diaries could clarify whether responders share identifiable physiological characteristics. Such data would help distinguish fermentable-carbohydrate sensitivity from visceral hypersensitivity, peripheral and central sensitization, bowel endometriosis, medication-related dysmotility, defecatory dysfunction, or other overlapping mechanisms. A negative response to a supervised restriction phase should lead to stopping the diet and reassessing the symptom pathway instead of intensifying avoidance. For current practice, a low-FODMAP diet may be cautiously considered when GI symptoms are prominent, particularly bloating, abdominal pain, altered stool pattern, constipation, diarrhea, urgency, or IBS-like complaints. It should be introduced as a trial of GI symptom management, ideally by a dietitian trained in FODMAP protocols and familiar with endometriosis-related dietary burden. The restrictive phase should be short, response should be assessed with predefined symptom targets, and continuation should depend on meaningful improvement. Reintroduction and personalization should follow to minimize unnecessary restriction and identify individual tolerance patterns. The diet should not be presented as a general “anti-endometriosis” diet or a substitute for gynecological care. Figure 2 summarizes the proposed clinical pathway: pre-intervention assessment, time-limited restriction with predefined response criteria, structured substrate-by-substrate reintroduction, long-term personalization, and continuous safeguards including cycle-aligned symptom recording, eating-disorder screening, and co-monitoring with gynecological care.

Several aspects of this review warrant caution. The search was focused on low-FODMAP and related fermentable-carbohydrate terms, so it was not designed to evaluate all dietary interventions in endometriosis. Only five clinical reports met the eligibility criteria, and their designs, populations, comparators, duration, and outcome instruments differed substantially. The inclusion of a case report and a retrospective audit was justified by the small evidence base, but these designs were interpreted descriptively. Publication bias is possible because small dietary studies with null findings may be less likely to appear in indexed literature.

## 5. Conclusions

The available evidence indicates that a low-FODMAP diet may reduce GI complaints in selected endometriosis patients, including bloating, abdominal pain, altered stool pattern, constipation, or IBS-like symptoms. The limited evidence base provides the strongest support for short-term restriction under controlled conditions, and weaker supporting evidence from real-world dietitian-guided pathways. Current data do not justify presenting the low-FODMAP diet as a “treatment” method for endometriosis itself. In clinical practice, FODMAP restriction should therefore be considered a time-limited, dietitian-guided GI symptom intervention, followed by structured reintroduction if clinical improvement occurs. Future studies should clarify patient selection, durability after reintroduction, and the nutritional and psychosocial consequences of longer-term use.

## Figures and Tables

**Table 1:** Characteristics of included primary clinical studies.

Study	Design and Setting	Population and Case Definition	Comparator	Follow-Up	Assessment Instruments
Varney et al., 2025 [39]	Single-blind randomized crossover feeding trial, Monash University, Melbourne; recruited via advocacy websites/social media.	35 randomized; 25 completed. Age ≥18 years to menopause. Prior laparoscopy and/or ultrasound verbally confirmed; reports not sighted. Rome criteria not required; baseline global GI VAS >30 mm.	Supplied nutritionally matched control diet modelled on Australian Dietary Guidelines (~20 g/day FODMAPs).	Two 28-day diet periods; ≥28-day washout; each period began on day 1 of cycle unless hormonally suppressed.	100 mm overall GI VAS; NIH PROMIS-GI; Bristol Stool Chart; GIQLI; EHP-30/EHP-23; DASS-21.
Keukens et al., 2025 [40]	Single-centre prospective cohort pilot, Máxima Medical Center, Veldhoven, Netherlands.	47 consented; 34 started; 24 completed. Premenopausal adults with endometriosis diagnosed by examination, ultrasound, MRI, or laparoscopy and debilitating bowel symptoms. Completers: 37.5% anamnestic IBS; 58.3% Rome III IBS.	Within-subject comparison with pre-intervention baseline.	≥14 weeks: 4-week elimination plus ≥10-week patient-variable reintroduction.	Groningen-DeFeC/Agachan Constipation Scoring System; EHP-30; post-diet bowel/pain questions; self-reported adherence.
van Haaps et al., 2023 [41]	Prospective one-centre patient-preference pilot with control group, Amsterdam UMC Endometriosis Center.	Per-protocol *n* = 62: low-FODMAP 22, endometriosis diet 21, control 19. Diagnosis by imaging, laparoscopy, or both. Eligible with VAS pain ≥3/10 for dysmenorrhea, deep dyspareunia, or chronic pelvic pain despite medical treatment.	No-diet usual-care control; endometriosis diet as second active diet arm.	6 months: 3 months dietitian-guided support plus 3 months independent continuation.	0–10 cm VAS for dysmenorrhea, deep dyspareunia, chronic pelvic pain, dysuria, bloating, tiredness; EHP-30; GIQLI; adherence/satisfaction.
Moore et al., 2017 [42]	Retrospective audit of prospectively collected data, private community IBS clinic, Christchurch, New Zealand.	160 women with Rome III IBS returning for follow-up: 59 with laparoscopically diagnosed endometriosis; 101 with no known endometriosis. Endometriosis based on referral/history/questionnaire and consultant gynecologist’s laparoscopy.	Same-clinic IBS group without known endometriosis, taught the same low-FODMAP diet.	First follow-up at 4 weeks; later follow-up as clinically required.	Practice 40-item questionnaire; response ≥50% abdominal symptom reduction; subjective adherence by direct questioning.
Jankovich and Watkins, 2017 [38]	Single-patient case report, private dietetic practice, South Africa; structured 16-week programme with individual and group sessions.	23-year-old woman with gynecologist-diagnosed IBS and ovarian endometriosis treated surgically by laparoscopy 4 months earlier. Persistent lower abdominal pain and bowel symptoms, worse during menses; coeliac disease excluded by negative tTG IgA on gluten-containing diet.	Within-patient comparison with pre-intervention baseline.	2-week first-line diet/lifestyle advice; 6-week strict low-FODMAP elimination including lactose; 10-week structured reintroduction; final personalization.	Practice 10-point symptom scale; Bristol Stool Form Chart; food/symptom diary; FODMAP challenge tolerance.

**Table 2:** Low-FODMAP implementation, dietitian involvement, and adherence.

Study	Low-FODMAP Protocol	Dietitian Involvement and Materials	Food Provision	Reintroduction/ Personalization	Adherence, Completion, Dropout
Varney et al., 2025 [39]	28-day low-FODMAP diet (<5 g/day) vs. control (~20 g/day). Both Australian-Dietary-Guidelines-based and matched for energy, macronutrients, fibre, gluten exposure, and low lactose.	Research dietitians designed diets; 7-day rotating meal plan; intake recording; diet names masked.	Most food centrally cooked, vacuum-packed in plain packaging, frozen, and delivered free of charge.	None; restriction phase only.	Daily adherence: 75% low-FODMAP days vs. 71% control. 35 randomized; 25 completed. Dropouts: 2 diet 1, 2 washout, 6 diet 2.
Keukens et al., 2025 [40]	4-week strict elimination, then ≥10-week reintroduction; minimum total duration 14 weeks.	Trained FODMAP dietitian: initial consult, handouts/recipes/tips/meal plans, phone monitoring during elimination, post-elimination follow-up, multiple reintroduction consults; costs covered.	No supplied meals.	FODMAPs reintroduced one at a time; timing adapted to symptom flares, lifestyle, and preferences. Completion required elimination plus reintroduction.	47 consented; 13 withdrew pre-start, mainly motivation/time or intervening surgery. 34 started; 24 completed. Completers: mean non-adherence 8%; 50% continued diet after study.
Van Haaps et al., 2023 [41]	Self-selection to low-FODMAP, endometriosis diet, or no diet. Low-FODMAP arm: elimination, reintroduction, personalization; two consultations per phase during 3-month guided period.	Dietitian in training supervised by registered dietitian; three 1 h and three 30 min consultations; food diaries before first consult.	No supplied meals. Low-FODMAP materials: weekly menus, nutrient/portion checklists, grocery lists, and practical food guide.	Three-phase low-FODMAP pathway during guided period; then 3 months of independent continuation.	Low-FODMAP PP *n* = 22. Three of 43 diet participants discontinued for personal reasons; no crossover. 81.8% of low-FODMAP arm wanted partial continuation at 6 months.
Moore et al., 2017 [42]	Low-FODMAP diet taught as primary therapy to all women with Rome III IBS in the clinic.	One-to-one teaching by IBS nurse specialist trained by experienced dietitian; 1-week food/symptom diary before instruction.	No supplied meals; Monash low-FODMAP app recommended and booklet provided.	Further instruction on gradual, systematic reintroduction according to individual responses.	Adherence by direct questioning: endometriosis 55/59 (93%); IBS without known endometriosis 91/101 (90%). Only follow-up returners included.
Jankovich and Watkins, 2017 [38]	Stepwise pathway: first-line IBS diet/lifestyle advice, then 6-week strict low-FODMAP elimination including lactose, followed by re-challenge.	Two individual and two group sessions over 16 weeks: 3 h group training, 3-week individual follow-up, 2 h re-challenge group, email support, final consult.	No supplied meals. Education: labels, menus, recipes, shopping, fibre, eating out, sauces, and nutritional adequacy.	10-week challenges: fructose, sorbitol, mannitol, wheat/onion/garlic fructans, GOS, lactose. Full-dose tolerance: lactose, sorbitol, GOS; no tolerance: mannitol, garlic fructans; half-dose: fructose, wheat fructans; initial dose: onion fructans. Final modified long-term diet.	Single patient completed. No formal adherence %. Symptoms controlled 1 month after final consult, with occasional constipation/bloating linked to identifiable meals.

**Table 3:** GI outcomes.

Study	GI Outcome Definition/Instrument	Global GI Response	Bloating/Abdominal Distension	Stool Pattern/Constipation/Diarrhea	Interpretive Constraints
Varney et al., 2025 [39]	Primary responder outcome: 100 mm overall GI VAS; responder ≥20 mm improvement from baseline to diet end and/or end-of-diet VAS <30 mm. Individual symptoms: PROMIS-GI/daily VAS; stool: Bristol.	Responders: ITT 21/35 (60%) vs. 9/35 (26%), *p* = 0.008; PP 18/25 (72%) vs. 8/25 (32%), *p* = 0.01. Week-4 overall GI symptoms: 35 vs. 58 mm, *p* < 0.001.	PROMIS bloating T-score improved vs. baseline and control; final-week daily VAS also lower on low-FODMAP.	Normal stool form: 71% of low-FODMAP days vs. 43% baseline and 56% control. Loose stools less frequent vs. baseline/control; PROMIS diarrhea improved vs. control; constipation improved vs. baseline and was nominally lower than control.	Small single-centre trial; single blinding not formally assessed; subjective outcomes; no mechanistic biomarkers; supplied restriction phase only, not real-world reintroduction/personalization.
Keukens et al., 2025 [40]	Primary outcome: post-reintroduction constipation by Groningen-DeFeC-derived Agachan score (0–30). Bloating by Groningen-DeFeC and post-diet questions.	84% of completers reported decreased bowel symptoms.	Binary bloating prevalence not significantly changed: 24/24 baseline, 15/19 after elimination, 21/21 after reintroduction; transient post-elimination decrease *p* = 0.125. Post-diet, 53% of completers reported less bloating.	Constipation score decreased from median of 7.0 (IQR 5) to 5.0 (IQR 4); MD 2.1, 95% CI 0.4–3.7, *p* = 0.023.	No control; completer analysis; high pre-/post-start dropout; self-reported data; listwise deletion. Binary bloating and subjective post-diet improvement are distinct outcomes.
van Haaps et al., 2023 [41]	0–10 cm VAS for bloating, dysuria, tiredness, and pain symptoms; GIQLI included, without between-group difference.	No global low-FODMAP GI responder endpoint.	Pooled diet arms vs. control: less bloating over 6 months, MD −0.84, 95% CI −1.68 to −0.004, *p* = 0.049. Low-FODMAP arm: baseline improvement *p* < 0.001, but vs. control not significant, MD −0.69, 95% CI −1.66 to 0.27, *p* = 0.159.	No constipation-specific outcome. Dysuria improved within low-FODMAP arm, *p* = 0.015.	Non-randomized patient-preference design; active diet arms pooled in several comparisons; limited low-FODMAP-specific between-group effects; no sample size calculation; no follow-up beyond 6 months.
Moore et al., 2017 [42]	Response ≥50% reduction in abdominal symptoms at 4-week follow-up; non-validated 40-item clinical questionnaire.	Response: endometriosis + IBS 43/59 (72%) vs. IBS without known endometriosis 49/101 (49%); *p* = 0.001, OR 3.11, 95% CI 1.5–6.2.	Individual bloating response NR.	Individual stool/constipation/diarrhea responses NR. Baseline diarrhea-predominant phenotype was less common in the endometriosis subgroup.	Retrospective audit; no placebo/control diet; only returners; comparator was IBS without known endometriosis, not systematically screened endometriosis-negative; non-validated tool; individual symptom changes not collected.
Jankovich and Watkins, 2017 [38]	Practice 10-point scale for bloating, abdominal pain, wind/flatulence, gurgling, constipation, nausea; Bristol Stool Form Chart.	Symptoms improved after first-line advice and further after strict low-FODMAP elimination; after 6-week elimination, symptoms were absent or ≤3/10.	Bloating 8/10 at baseline, persisted after first-line advice, improved after elimination; occasional recurrence during maintenance linked to specific meals/ingredients.	Baseline bowel movements every 2–3 days, Bristol type 1–2; final follow-up every 1–2 days, type 3. Occasional laxative use persisted.	Single case; no control; practice scale; concomitant lifestyle, laxative, and stress changes. Lactose: failed informal home self-test, but structured clinical re-challenge showed full-dose tolerance; lactose intolerance not confirmed.

IBS, irritable bowel syndrome; FODMAP, fermentable oligosaccharides, disaccharides, monosaccharides, and polyols; EHP-30, Endometriosis Health Profile-30; GIQLI, Gastrointestinal Quality of Life Index; VAS, visual analogue scale; ITT, intention-to-treat; PP, per-protocol; QoL, quality of life; MD, mean difference.

**Table 4:** Pain, quality-of-life, acceptability, and safety outcomes.

Study	Pain Outcomes	Endometriosis-Specific QoL	GI-Related QoL/Psychological Outcomes	Acceptability and Safety	Limits for Interpretation
Varney et al., 2025 [39]	EHP-30 pain improved on low-FODMAP vs. control: median 22.7 vs. 40.9, *p* = 0.005. This was an EHP-30 domain; pelvic pain, dysmenorrhea, and dyspareunia were not separate symptom endpoints.	EHP-30 total better on low-FODMAP vs. control: 45.3 vs. 47.1, *p* = 0.028. Control/powerlessness nominally improved vs. control, *p* = 0.045; EHP-23 work improved vs. baseline but not control.	GIQLI HRQOL improved vs. control: median 88 vs. 81, *p* = 0.003; symptoms and physical function improved. DASS-21 depression/anxiety did not differ in full ITT; among abnormal baselines, depression decreased and anxiety trended down. Stress decreased vs. baseline without diet-specific difference in abnormal-stress subgroup.	Serious AE: appendicitis during washout after control diet, unlikely related. Two gastroenteritis-like events during control diet; one later withdrew citing life stress.	Strong feeding-control design, but endometriosis-specific pain was not a primary symptom endpoint. Results apply to short-term supplied restriction.
Keukens et al., 2025 [40]	EHP-30 pain improved from 47.8 +/− 20.1 to 29.2 +/− 17.3, *p* = 0.002; 65% of completers reported less pain, especially chronic pelvic pain.	Post-reintroduction EHP-30 improvements: control/powerlessness 69.4 to 36.7, *p* < 0.001; emotional well-being 45.2 to 29.2, *p* = 0.001; social support 46.4 to 31.3, *p* = 0.017; self-image 51.2 to 40.5, *p* = 0.035; work life 35.0 to 21.7, *p* = 0.003; sexual intercourse 61.6 to 45.7, *p* = 0.023.	No GIQLI; QoL captured by EHP-30 domains.	63% of starters agreed diet was easy to maintain; 87% saw it as a good addition to therapy; 90% would recommend it. No harmful effects reported in discussion.	Completer-only interpretation; 13/47 withdrew pre-start and 10/34 post-start. Improvements may reflect a motivated, responder-enriched subgroup.
van Haaps et al., 2023 [41]	Pooled diet arms improved deep dyspareunia, dysuria, bloating, and tiredness vs. baseline. Low-FODMAP arm improved dysuria (*p* = 0.015) and bloating (*p* < 0.001) from baseline. Separated low-FODMAP vs. control: less deep dyspareunia, MD −1.15, 95% CI −2.2 to −0.10, *p* = 0.032; dysmenorrhea and chronic pelvic pain not significant.	Pooled diet arms improved EHP-30 pain, powerlessness, emotional well-being, self-image, work life, and sexual intercourse from baseline. Vs control, pooled effects remained for social support and medical profession. Low-FODMAP vs. control improved medical profession, MD −17.14, *p* = 0.018; most separated-arm QoL effects were not significant.	GIQLI improved within diet groups but not vs. control in pooled diet analysis (*p* = 0.539); separated low-FODMAP vs. control also not significant (*p* = 0.511).	No side effects. 35/43 diet participants wanted at least partial continuation at 6 months; 81.8% in low-FODMAP arm.	Self-selection; pooled diet effects may not represent low-FODMAP-specific effects; low-FODMAP and endometriosis diet often not separable.
Moore et al., 2017 [42]	Pain variables were baseline phenotype descriptors, not post-diet endpoints; dyspareunia and referred pelvic/back pain were associated with endometriosis subgroup.	No EHP-30 or endometriosis-specific QoL instrument.	No GIQLI or psychological instrument.	High adherence by direct questioning; safety/adverse events NR as specific outcomes.	IBS service audit; post-diet outcome was bowel-symptom response, not gynecologic pain or QoL.
Jankovich and Watkins, 2017 [38]	Abdominal pain decreased from 7/10 at baseline to ≤3/10 after strict elimination. Dysmenorrhea, dyspareunia, and pelvic pain were not formally assessed.	No EHP-30 or validated endometriosis QoL instrument.	No GIQLI; report described better symptom control, trigger recognition, and self-management understanding.	No adverse effects reported; at 1-month follow-up, patient maintained a balanced modified diet with sufficient FODMAP restriction.	Case report with multiple concomitant interventions and no validated QoL endpoints.

## Data Availability

No new data were created or analyzed in this study. Data sharing is not applicable to this article.

## Supplementary Materials

The following supporting information can be downloaded at: https://www.mdpi.com/article/10.3390/nu18132164/s1: PRISMA 2020 Checklist.

## Abbreviations

ACOG, American College of Obstetricians and Gynecologists; BASE, Bielefeld Academic Search Engine; CI, confidence interval; DASS-21, Depression Anxiety Stress Scales-21; EHP-23/EHP-30, Endometriosis Health Profile-23/30; FODMAP, fermentable oligosaccharides, disaccharides, monosaccharides, and polyols; GI, gastrointestinal; GIQLI, Gastrointestinal Quality of Life Index; IBS, irritable bowel syndrome; ITT, intention-to-treat; MD, mean difference; MRI, magnetic resonance imaging; NR, not reported; OR, odds ratio; PP, per-protocol; PRISMA, Preferred Reporting Items for Systematic Reviews and Meta-Analyses; PROMIS-GI, Patient-Reported Outcomes Measurement Information System Gastrointestinal; QoL, quality of life; RoB 2, revised Cochrane risk-of-bias tool for randomized trials; ROBINS-I, Risk Of Bias In Non-randomized Studies of Interventions; VAS, visual analogue scale.

## Appendix A

**Table A1:** Examples of foods commonly considered higher in fermentable oligosaccharides, disaccharides, monosaccharides, and polyols (FODMAPs), together with commonly used lower-FODMAP alternatives [28,30,38,49].

FODMAP Category	Examples of Higher-FODMAP Foods	Lower-FODMAP Alternatives
Excess fructose	Apple, pear, mango, watermelon, honey	Banana, orange, kiwi, grapes, strawberries
Lactose	Cow’s milk, regular yogurt, soft cheese, ice cream	Lactose-free milk, lactose-free yogurt, hard cheeses, plant-based milk with low-FODMAP composition
Fructans	Wheat bread, wheat pasta, onion, garlic, rye products	Rice, oats, potatoes, quinoa, sourdough spelt bread in tolerated portions, chives, garlic-infused oil
Galacto-oligosaccharides (GOS)	Chickpeas, lentils, kidney beans, baked beans	Firm tofu, canned lentils in tolerated portions, quinoa, rice, small tolerated servings of lower-FODMAP protein accompaniments
Polyols	Stone fruits, apple, pear, mushrooms, cauliflower, products containing sorbitol or mannitol	Blueberries, grapes, pineapple, carrots, zucchini, cucumber, products without polyol sweeteners

**Table A2:** Risk of bias and quality assessment of included studies.

Study	Appraisal Tool	Main Strengths	Risk-of-Bias-Relevant Limitations	Overall Judgement
Varney et al., 2025 [39]	RoB 2, crossover RCT considerations	Randomized crossover design; prospective registration; nutritionally matched supplied diets; menstrual-cycle timing; validated GI/QoL instruments; prespecified VAS-based responder definition; power calculation; no evident order effects.	Single blinding only, not assessed; subjective outcomes; 10/35 dropout; Melbourne advocacy-channel recruitment; reports not sighted; Rome criteria not required; control FODMAP dose may exceed typical habitual intake; no biomarkers, reintroduction, or long-term follow-up.	Strongest evidence source, but small and short-term; supports restriction-phase proof-of-concept in selected women with poorly controlled GI symptoms.
Keukens et al., 2025 [40]	ROBINS-I/prospective single-arm cohort principles	Prospective registration; bowel-symptom eligibility; dietitian-led elimination/reintroduction; validated constipation score and EHP-30; detailed dropout reporting; real-world implementation.	No control, randomization, or blinding; high pre-/post-start attrition; completer-based efficacy; self-reported symptoms/adherence; listwise deletion; sample size increased after unexpected dropout; no long-term nutritional/microbiome safety data.	Useful real-world cohort in motivated completers; high risk of selection and attrition bias.
van Haaps et al., 2023 [41]	ROBINS-I/non-randomized controlled intervention	Prospective controlled design; no-diet control; imaging/laparoscopy diagnosis; 6-month follow-up; dietitian guidance; EHP-30/VAS outcomes; satisfaction and side effects reported.	Group self-selection; no sample size calculation; frequent pooling of low-FODMAP and endometriosis diet arms; no blinding/placebo diet; possible expectancy/motivation effects; limited low-FODMAP-specific estimates; no follow-up beyond 6 months.	Important controlled pilot, but limited low-FODMAP-specific causal inference; useful for dietitian-guided intervention and acceptability.
Moore et al., 2017 [42]	ROBINS-I/retrospective audit of prospectively collected clinical data	Prospectively entered clinical database; consecutive clinic setting; same teaching in both groups; explicit response threshold; adherence table; clinically relevant endometriosis-IBS subgroup observation.	Retrospective private-clinic audit; only returners; no placebo/control diet; non-validated questionnaire; subjective adherence; comparator not systematically screened and may include occult endometriosis; individual symptom changes not collected.	Hypothesis-generating; useful for identifying a candidate subgroup, weak for causal effect estimation.
Jankovich and Watkins, 2017 [38]	JBI case report checklist	Detailed clinical description; staged dietary process; structured FODMAP-subgroup reintroduction/tolerance mapping; stool form and symptom severity tracked; implementation detail.	Single patient; no control; non-validated practice scale; concurrent diet/lifestyle advice, laxative optimization, probiotic changes, and stress changes; no standardized endometriosis pain/QoL endpoints or effect estimate. Informal lactose self-test conflicted with supervised re-challenge; clinical re-challenge is definitive.	Descriptive only; valuable for reintroduction/personalization illustration, not population-level efficacy.

IBS, irritable bowel syndrome; FODMAP, fermentable oligosaccharides, disaccharides, monosaccharides, and polyols; EHP-30, Endometriosis Health Profile-30; GI, gastrointestinal; QoL, quality of life; VAS, visual analogue scale.
